# Comparative Genomics of Streptococcus oralis Identifies Large Scale Homologous Recombination and a Genetic Variant Associated with Infection

**DOI:** 10.1128/msphere.00509-22

**Published:** 2022-11-02

**Authors:** Luke R. Joyce, Madison A. Youngblom, Harshini Cormaty, Evelyn Gartstein, Katie E. Barber, Ronda L. Akins, Caitlin S. Pepperell, Kelli L. Palmer

**Affiliations:** a Department of Biological Sciences, The University of Texas at Dallas, Richardson, Texas, USA; b Microbiology Doctoral Training Program, University of Wisconsin-Madison, Madison, Wisconsin, USA; c Department of Medical Microbiology and Immunology, School of Medicine and Public Health, University of Wisconsin-Madison, Madison, Wisconsin, USA; d Department of Pharmacy Practice, University of Mississippi School of Pharmacy, University of Mississippi, Jackson, Mississippi, USA; e Methodist Charlton Medical Center, Dallas, Texas, USA; f Department of Medicine (Infectious Diseases), School of Medicine and Public Health, University of Wisconsin-Madison, Madison, Wisconsin, USA; University of Iowa

**Keywords:** *Streptococcus*, bacteremia, genomics, infective endocarditis

## Abstract

The viridans group streptococci (VGS) are a large consortium of commensal streptococci that colonize the human body. Many species within this group are opportunistic pathogens causing bacteremia and infective endocarditis (IE), yet little is known about why some strains cause invasive disease. Identification of virulence determinants is complicated by the difficulty of distinguishing between the closely related species of this group. Here, we analyzed genomic data from VGS that were isolated from blood cultures in patients with invasive infections and oral swabs of healthy volunteers and then determined the best-performing methods for species identification. Using whole-genome sequence data, we characterized the population structure of a diverse sample of Streptococcus oralis isolates and found evidence of frequent recombination. We used multiple genome-wide association study tools to identify candidate determinants of invasiveness. These tools gave consistent results, leading to the discovery of a single synonymous single nucleotide polymorphism (SNP) that was significantly associated with invasiveness. This SNP was within a previously undescribed gene that was conserved across the majority of VGS species. Using the growth in the presence of human serum and a simulated infective endocarditis vegetation model, we were unable to identify a phenotype for the enriched allele in laboratory assays, suggesting a phenotype may be specific to natural infection. These data highlighted the power of analyzing natural populations for gaining insight into pathogenicity, particularly for organisms with complex population structures like the VGS.

**IMPORTANCE** The viridians group streptococci (VGS) are a large collection of closely related commensal streptococci, with many being opportunistic pathogens causing invasive diseases, such as bacteremia and infective endocarditis. Little is known about virulence determinants in these species, and there is a distinct lack of genomic information available for the VGS. In this study, we collected VGS isolates from invasive infections and healthy volunteers and performed whole-genome sequencing for a suite of downstream analyses. We focused on a diverse sample of Streptococcus oralis genomes and identified high rates of recombination in the population as well as a single genome variant highly enriched in invasive isolates. The variant lies within a previously uncharacterized gene, *nrdM*, which shared homology with the anaerobic ribonucleoside triphosphate reductase, *nrdD*, and was highly conserved among VGS. This work increased our knowledge of VGS genomics and indicated that differences in virulence potential among S. oralis isolates were, at least in part, genetically determined.

## INTRODUCTION

The viridans group streptococci (VGS) comprise a diverse collection of alpha and nonhemolytic streptococci that inhabit the oral cavity and gastrointestinal and genitourinary tracts of healthy humans (1). VGS are also associated with invasive disease, particularly in immunocompromised hosts, and are estimated to cause ~23% of Gram-positive bacteremia (2, 3) and ~17% of infective endocarditis (IE) cases (4). Bacterial determinants of invasiveness among the VGS are not well understood. Research in this area is hampered by the fact that the specific species of VGS causing bacteremia and IE are infrequently determined in a clinical context due to the lack of resolution of existing diagnostic microbiological tools (5, 6). Vitek2 and MicroScan allow for the general assignment of isolates to VGS, and the assignment of a limited number of VGS species specifically. Even a relatively newer technique in clinical diagnostics, matrix-assisted laser desorption ionization-time of flight mass spectrometry (MALDI-TOF MS), fails to resolve certain VGS species, including Streptococcus mitis and Streptococcus oralis (7). 16S rRNA gene sequencing, multilocus sequence analysis, other genotyping schemes (5–10), and GyrB typing (11) are commonly used methods for molecular VGS identification but are not generally employed in clinical laboratories.

Retrospective studies using molecular approaches have determined that in addition to being present as oral commensals in healthy individuals, S. mitis and S. oralis stand out among VGS as major causative agents of VGS bacteremia and IE (8, 12–15). S. mitis and S. oralis are members of the mitis group streptococci, a subgroup within the VGS that is closely related to the major human pathogen Streptococcus pneumoniae (16, 17). A recent study in oncology patients demonstrated that S. mitis (58%) and S. oralis (19%) were the most frequently identified species in VGS infections over ~1.5 years (8). While S. mitis and S. oralis are a significant burden on immunocompromised patients, the mechanisms of virulence within these species have not been fully elucidated. More specifically, it is not known whether all members of these species have equal pathogenic potential, or whether some strains have a higher propensity for causing invasive disease than others.

In this study, we aimed to investigate the mechanisms of invasiveness among VGS by characterizing species diversity of presumptive VGS obtained clinically from bacteremia and endocarditis patients. Clinical isolate genomes obtained in this study were supplemented by existing genome sequences with curated metadata in public databases and genome sequences of oral isolates collected from healthy volunteers. Our results supported metagenomic sequence binning as a high-resolution tool for differentiating species within the VGS grouping. After a preliminary analysis in which we determined species designations for clinical isolates that were diagnosed as VGS, we focused our study on S. oralis as a prominent cause of invasive infection. Within a large sample of S. oralis isolates from all three described subspecies (18) (subsp. *dentisani*, subsp. *tigurinus*, and subsp. *oralis*) we found high levels of diversity and strikingly high recombination rates. We used multiple genome-wide association study (GWAS) methods to test the hypothesis that specific genetic variations were associated with invasive infection (compared to commensal isolates) among S. oralis isolates. We discovered a SNP in a previously uncharacterized gene that was significantly enriched in invasive isolates compared to noninvasive isolates. The contribution of this novel locus to growth with human serum and in a simulated infective endocarditis vegetation model (19) was assessed, although we were unable to identify a phenotype for a gene knockout or allele of the significant variant under the conditions tested. This work (i) increased the genomic information available for VGS strains, (ii) described population structure and large-scale homologous recombination within the S. oralis species, and (iii) provided evidence that the propensity for virulence in S. oralis was at least in part genetically determined.

## RESULTS

### GyrB typing and Kraken efficiently identified S. mitis and S. oralis.

VGS isolates from the Dallas, Texas and Jackson, Mississippi areas were collected from clinically confirmed VGS bacteremia and infective endocarditis patients. Isolates were initially characterized using either Vitek2 or MicroScan platforms. A total of 66 clinical isolates were successfully subcultured in the laboratory. However, two isolates were incorrectly identified as Streptococcus spp., one Enterococcus faecalis and one Aerococcus urinae strain, resulting in 64 presumptive VGS strains collected (Table S1). To compare isolates causing invasive disease against commensal isolates, healthy volunteers were recruited for oral swab collection on the University of Texas at Dallas campus for a total of 81 VGS isolates (Table S1).

10.1128/msphere.00509-22.7 TABLE S1 Newly sequenced VGS isolates. Download Table S1, XLSX file, 0.02 MB. Copyright © 2022 Joyce et al. 2022 Joyce et al. https://creativecommons.org/licenses/by/4.0/ This content is distributed under the terms of the Creative Commons Attribution 4.0 International license.

**FIG 1:**
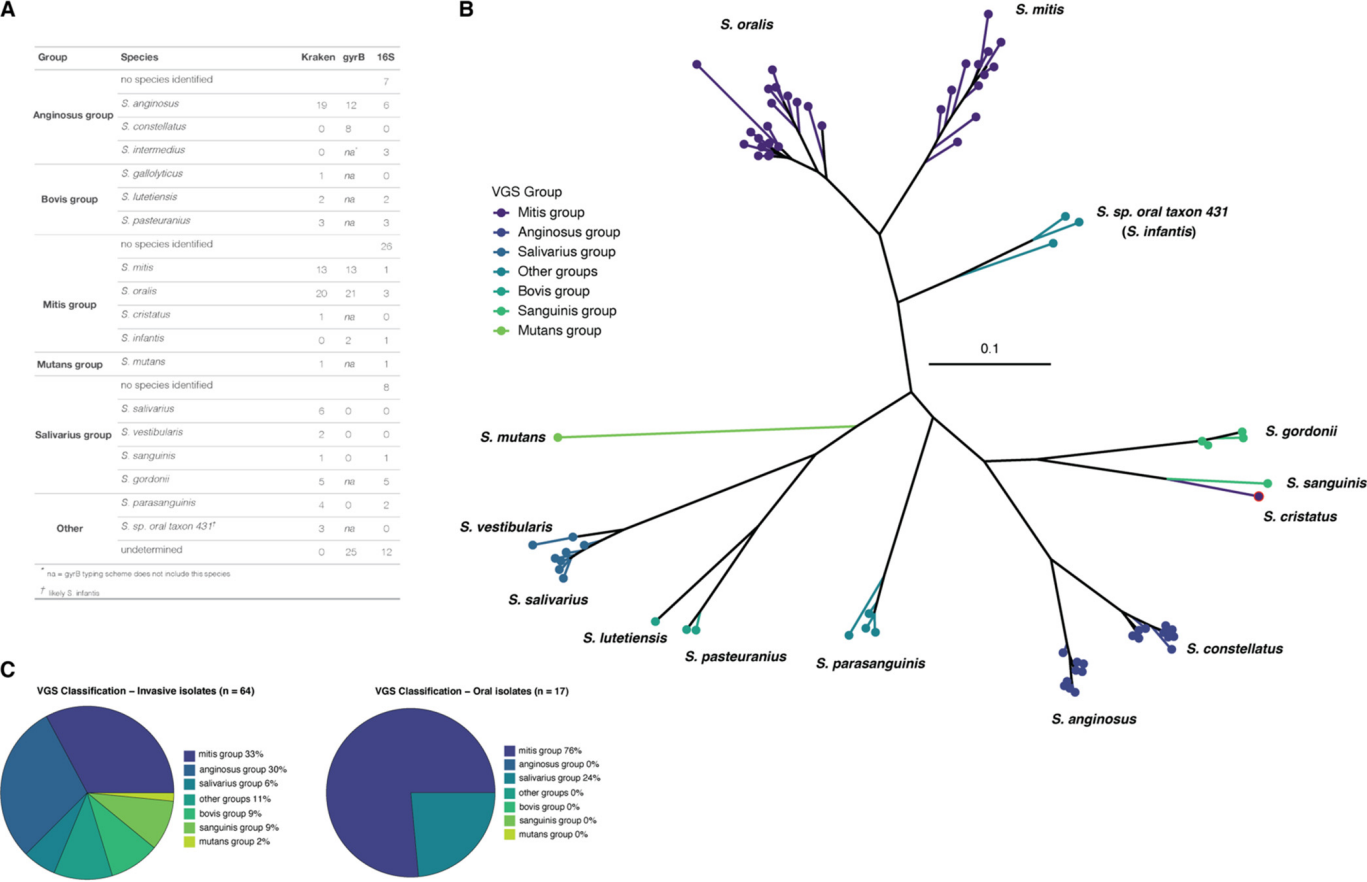
Species identification of blood and oral commensal viridans group streptococci (VGS). (A) Three different methods for species identification were used (Kraken, GyrB typing, and 16S rRNA gene sequencing), providing differing results. “na” values in the gyrB column indicate species not included in this typing scheme. “No species identified” indicates that the isolate could only be identified at the group level. (B) The phylogenetic tree of all 81 isolates was inferred from an alignment of GyrB sequences and identified by the VGS group and species as assigned by Kraken. The isolate which appears to have been misidentified by Kraken is outlined in red. (C) Invasive (left) and oral (right) isolates by VGS group according to species identification performed by Kraken.

As has previously been reported, clinical methods of VGS species identification (Vitek2 and MicroScan) were not effective for delineating closely related species, particularly within the mitis group. However, they were almost always correct in identifying an isolate as a part of the VGS (Table S1). The taxonomy of the clinical and oral isolates was analyzed by 16S rRNA gene sequencing, GyrB typing (11), and analysis of Illumina whole-genome sequencing (WGS) data using Kraken (20). While we expected that WGS would provide the clearest results, we also wanted to assess the utility of the GyrB typing scheme for a diverse set of VGS species because this method was less expensive, faster, and more feasible for researchers without computational experience and resources. Using these three different methods of species identification, we classified isolates into VGS groups using the taxonomy described by Facklam (21). All six of the major VGS groups (1) were represented in our sample of 81 isolates, with two isolates not fitting into any of these six groups (Fig. 1A). 16S rRNA sequencing was frequently unable to provide resolution to the species level but was often able to identify which group the isolate belonged to. GyrB typing (11) and Kraken (20) were both effective in distinguishing S. oralis and S. mitis. However, GyrB typing was not generally accurate for other VGS groups (Fig. 1A).

One downside of species identification with Kraken was it seemed unable to distinguish between anginosus group isolates. S. anginosus and S. constellatus were species within the anginosus group (also known as the milleri group) of the VGS and were recognized as abscess-causing bacteria, with more recent data suggesting their emergence as uropathogenic (1, 22, 23). Kraken identified 19 S. anginosus isolates. By GyrB typing, a total of 20 anginosus group isolates were identified (12 S. anginosus and 8 S. constellatus). These data suggested that S. mitis and S. oralis were accurately identified with either Kraken or GyrB typing, yet anginosus group species may be better identified by GyrB typing.

One drawback to species identification with GyrB typing was the current scheme was limited in the species it could distinguish (Fig. 1A). To further assess the relative functionality of these two methods, we inferred a phylogenetic tree from GyrB sequences and mapped species as defined by Kraken onto the tree (Fig. 1B). The added benefit of a phylogeny of GyrB sequences was the visualization of additional species not included in the typing scheme (e.g., species in the bovis group; Fig. 1B), which allowed us to confidently assign species to most isolates when combined with Kraken output. There was only a single instance where Kraken may have misidentified the species. An isolate identified by Kraken as S. cristatus (a member of the mitis group) appeared to cluster with S. sanguinis isolates (of the sanguinis group) on the GyrB phylogeny (Fig. 1B). Additionally, combining data from Kraken and GyrB typing showed that 2 out of the 3 isolates identified as *S.* sp. *oral taxon 431* by Kraken were identified as S. infantis (a member of the mitis group) by GyrB typing, and all 3 clustered together on the GyrB phylogeny, indicating that S. infantis was likely the correct designation (Fig. 1B).

Overall, our data show that closely related S. mitis and S. oralis can be identified accurately via either Kraken or GyrB typing. However, anginosus group isolates were better distinguished by GyrB typing. For data sets suspected to contain a diverse sample of different VGS species, species identification using Kraken with whole-genome sequence data appears the most robust. GyrB typing was an accurate method for making distinctions within species subsets, and the phylogeny of GyrB sequences may also provide additional information. Our results support previous assertions that 16S rRNA sequencing was not an effective method for distinguishing the closely related species of the VGS (1).

When we separated our samples into isolates from blood cultures and those from the mouths of healthy persons, we saw that invasive isolates spanned all 6 groups while isolates from oral isolates were less diverse and only originated from the mitis and salivarius groups (Fig. 1C). The lack of diversity in the oral isolates was due to the use of Mitis-Salivarius agar, which inhibits Gram-negative and most Gram-positive bacterial growth due to the presence of inhibitory nutrients. However, S. mitis, S. salivarius, and enterococci will still grow and produce different colony morphologies to allow preferential selection (24). It has been reported that S. mitis is the predominant species found in healthy oral microbiomes (25–30) with other commonly found VGS species, including S. oralis (27, 28), S. sanguinis (28, 29), and S. salivarius (29).

### High diversity among commensal and invasive S. oralis strains.

**FIG 2:**
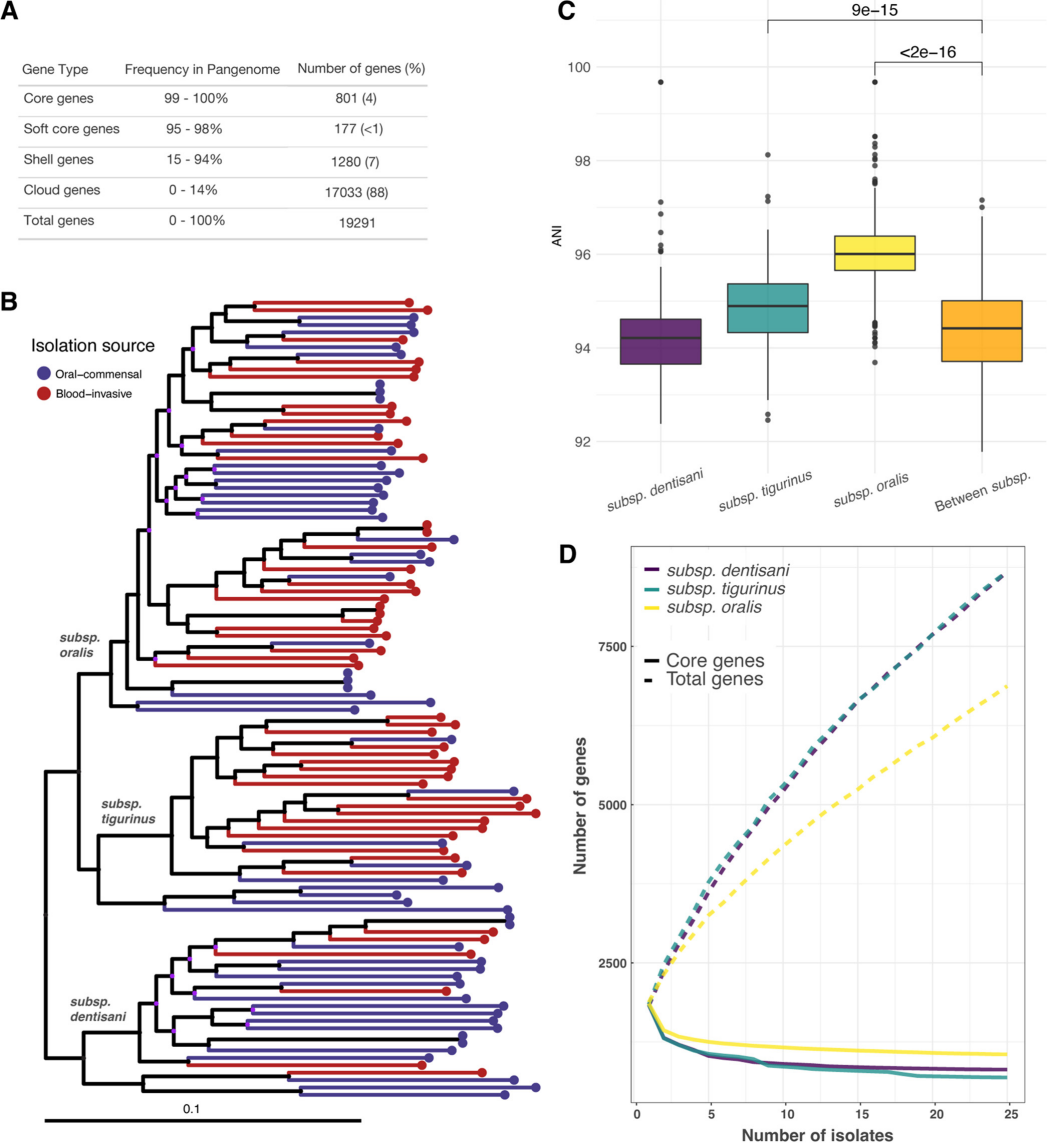
Pangenome analysis of S. oralis strains. (A) Summary statistics from pangenome analysis of all S. oralis GWAS isolates (*n* = 108). (B) Core genome phylogeny of S. oralis isolates inferred using RAxML and midpoint rooted. Tips colored by isolation source (oral-commensal in blue, blood-invasive in red). A total of 108 isolates were included with 57 isolated from the oral cavity and 51 isolated from invasive infections. Three subspecies are labeled, and the scale bar is given in SNPs per site. Nodes colored in purple represent bootstrap values <50. (C) Average nucleotide identity (ANI) calculated within and between the three subspecies of S. oralis phylogeny showed relatively low sequence conservation (~94% to 96% ANI) between even strains of the same subspecies. ANI values within subsp. *oralis* and subsp. *tigurinus* showed significantly higher sequence conservation within compared to between subspecies (Mann-Whitney U test with Benjamini-Hochberg correction). (D) Accumulation and rarefaction curves for three subspecies. All samples were repeatedly subsampled to the size of the smallest sample (subsp. *dentisani*, *n* = 25), and the median core and total gene values were plotted for 100 iterations.

S. oralis was the predominant single species (20/81 isolates) in our sample. To identify possible genetic variants associated with invasive infection, we gathered a larger sample of S. oralis. We obtained all isolates labeled as S. oralis and S. mitis from NCBI (see Materials and Methods for inclusion criteria) and used Kraken, a core genome phylogeny, and accessory gene content to confirm S. oralis isolates and identify mislabeled isolates. We found that a core genome alignment produced by Roary (31) effectively delineated S. oralis from S. mitis, as did patterns of accessory gene content (Fig. S1). We were able to identify 11 isolates mislabeled as S. mitis within NCBI databases, which we identified as S. oralis using these methods (Table S2). We ended up with a total of 108 S. oralis isolates, 57 oral commensals, and 51 from invasive infections (including bacteremia and IE) that we referred to as “oral” and “blood” isolates, respectively (Table S2). After assembly and annotation of all genomes, we performed a pangenome analysis with Roary and found that our samples had a core genome of only 801 genes, with an average genome size of 1898 genes. This meant that a significant proportion of genes (57%) encoded by an individual isolate were variable accessory genes (Fig. 2A). The accessory gene content in our sample of S. oralis isolates was highly diverse, with 88% of all genes found in our sample at a frequency of 14% or less and >19,000 genes identified in the pangenome (Fig. 2A). This high level of gene content diversity was mirrored in the core genome phylogeny, which has long terminal branch lengths indicating high variability between core genome sequences (Fig. 2B). Isolates from commensal and invasive sources were interspersed on the phylogeny and did not generally form monophyletic clades nor were the commensal or invasive phenotypes associated any subclades within the phylogeny (Fig. 2B).

10.1128/msphere.00509-22.1 FIG S1 Core genome phylogeny of all S. oralis and S. mitis isolates (from newly sequenced samples and NCBI) alongside a matrix of gene content. Clear delineation between S. oralis and S. mitis can be seen both in the core genome sequence as well as in core and accessory gene content differences between the species. Pangenome analysis was performed using Roary and visualization with Phandango. Download FIG S1, PDF file, 0.1 MB. Copyright © 2022 Joyce et al. 2022 Joyce et al. https://creativecommons.org/licenses/by/4.0/ This content is distributed under the terms of the Creative Commons Attribution 4.0 International license.

10.1128/msphere.00509-22.8 TABLE S2 S. oralis GWAS Strains. Download Table S2, XLSX file, 0.02 MB. Copyright © 2022 Joyce et al. 2022 Joyce et al. https://creativecommons.org/licenses/by/4.0/ This content is distributed under the terms of the Creative Commons Attribution 4.0 International license.

We identified three subclades within our core genome phylogeny (Fig. 2B) that corresponded with the three previously described subspecies of S. oralis, subsp. *dentisani*, subsp. *tigurinus*, and subsp. *oralis* (18), by cross-referencing our tree with the subspecies of some of the publicly available isolates in our data set (Table S2). Average nucleotide identity (ANI) values for core genome sequences within each subspecies show that subsp. *oralis* was the most conserved, followed by subsp. *tigurinus* and subsp. *dentisani* (Fig. 2C). Subsp. *dentisani* was unique in that it had ANI values resembling those measured between isolates of different subspecies. This indicated that the level of diversity within this subspecies was similar to those found between subspecies (Fig. 2C). Following the trends we identified in core genome diversity levels, the pangenome of subsp. *oralis* isolates appeared more conserved (more core genes, fewer accessory genes) than either subsp. *tigurinus* or subsp. *dentisani* (Fig. 2D). Higher core genome and pangenome diversity levels among subsp. *tigurinus* and subsp. *dentisani* isolates could indicate that these isolates have access to more diverse partners for horizontal gene transfer (HGT).

### High levels of recombination among S. oralis isolates.

**FIG 3:**
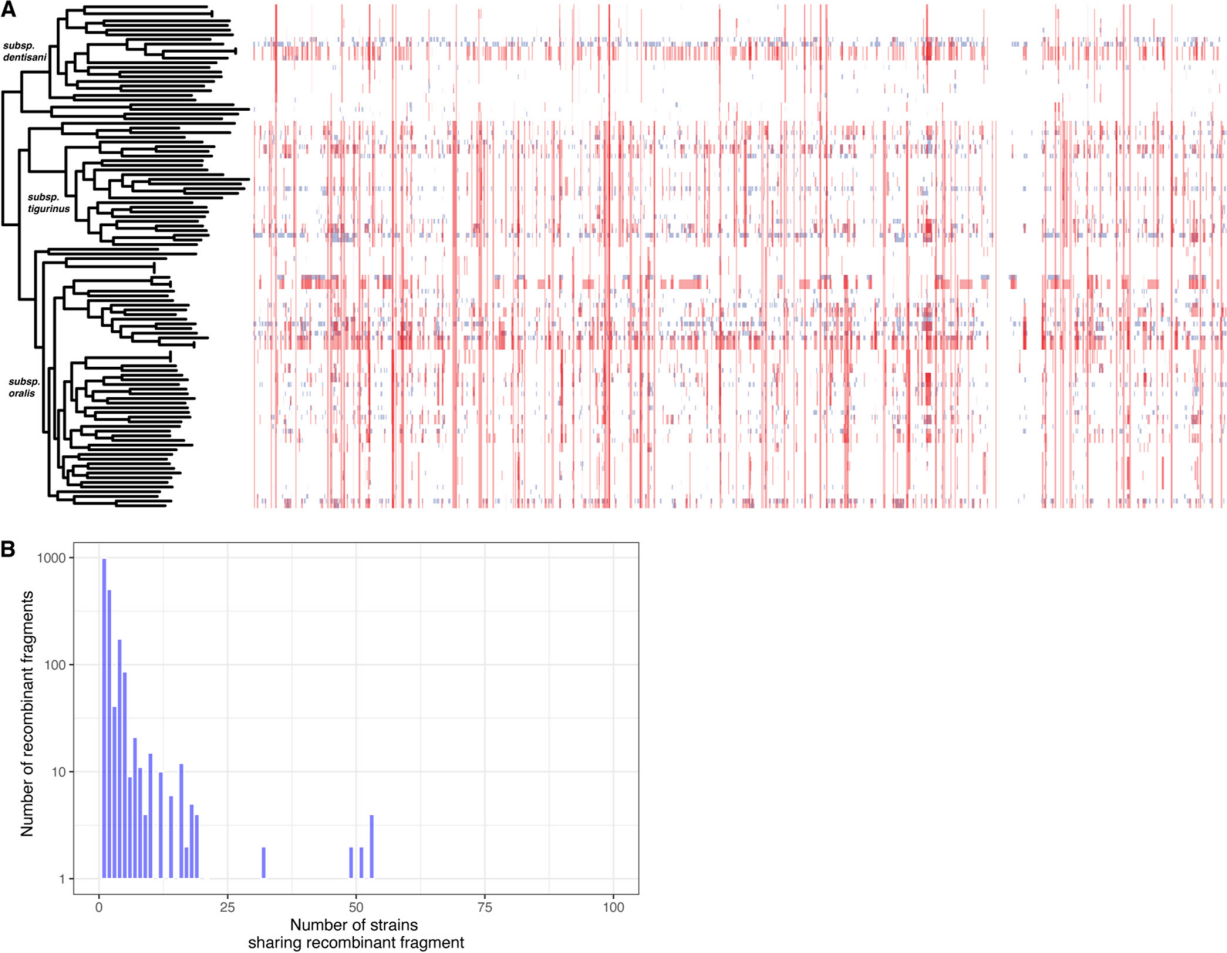
S. oralis strains were exceptionally recombinogenic. (A) Recombination tracts predicted by Gubbins in the core genome of S. oralis isolates plotted alongside the core genome phylogeny. Red tracts represent recombination within the sample and blue tracts represent recombination with isolates outside this sample. In total, 99.9% of the core genome had been affected by recombination. Visualization was performed with Phandango. (B) Histogram showing the number of strains in our sample that shared a given recombinant fragment. The *x*-axis is the number of strains that shared a recombinant fragment (with a maximum of 108 isolates, the size of our sample) and the *y*-axis is the number of fragments identified by Gubbins that were shared by that number of isolates.

Viridans group streptococci (VGS) were known for being naturally competent (32) and for evolving rapidly via widespread homologous recombination (33, 34). We characterized the signatures of recombination in the core genome of S. oralis using Gubbins (35) and identified extreme amounts of recombination, where 99.9% of the core genome was within a predicted recombinant fragment in at least one isolate (Fig. 3A). Additionally, we noted that recombinant fragments were not shared across multiple isolates but were usually present in only a few isolates (Fig. 3B). Using ClonalFrameML (36) we calculated the *r/m* value – the ratio of SNPs imported via recombination (r) to those introduced randomly (m) – and found that with an *r/m* of 5.77. The genetic diversity in our sample was ~6× more likely to be introduced via recombination. This was slightly less than the notoriously recombinogenic S. pneumoniae (*r/m* = ~7) (33) but much higher than other IE-causing bacteria, such as S. aureus (*r/m* = <1) (37).

### Genome-wide association study revealed that the variant was associated with invasiveness.

With a large sample balanced between our phenotypes of interest, we used multiple genome-wide association study (GWAS) methods to identify genetic variants associated with invasive disease, which included isolates from both IE and bacteremia patients. We started by looking for associations between accessory genes and invasiveness using Scoary (38), which yielded no significant results. This was perhaps not surprising given that accessory gene content was diverse in the sample and individual accessory genes were, thus, unlikely to be shared by a large proportion of isolates (Fig. 1A).

**FIG 4:**
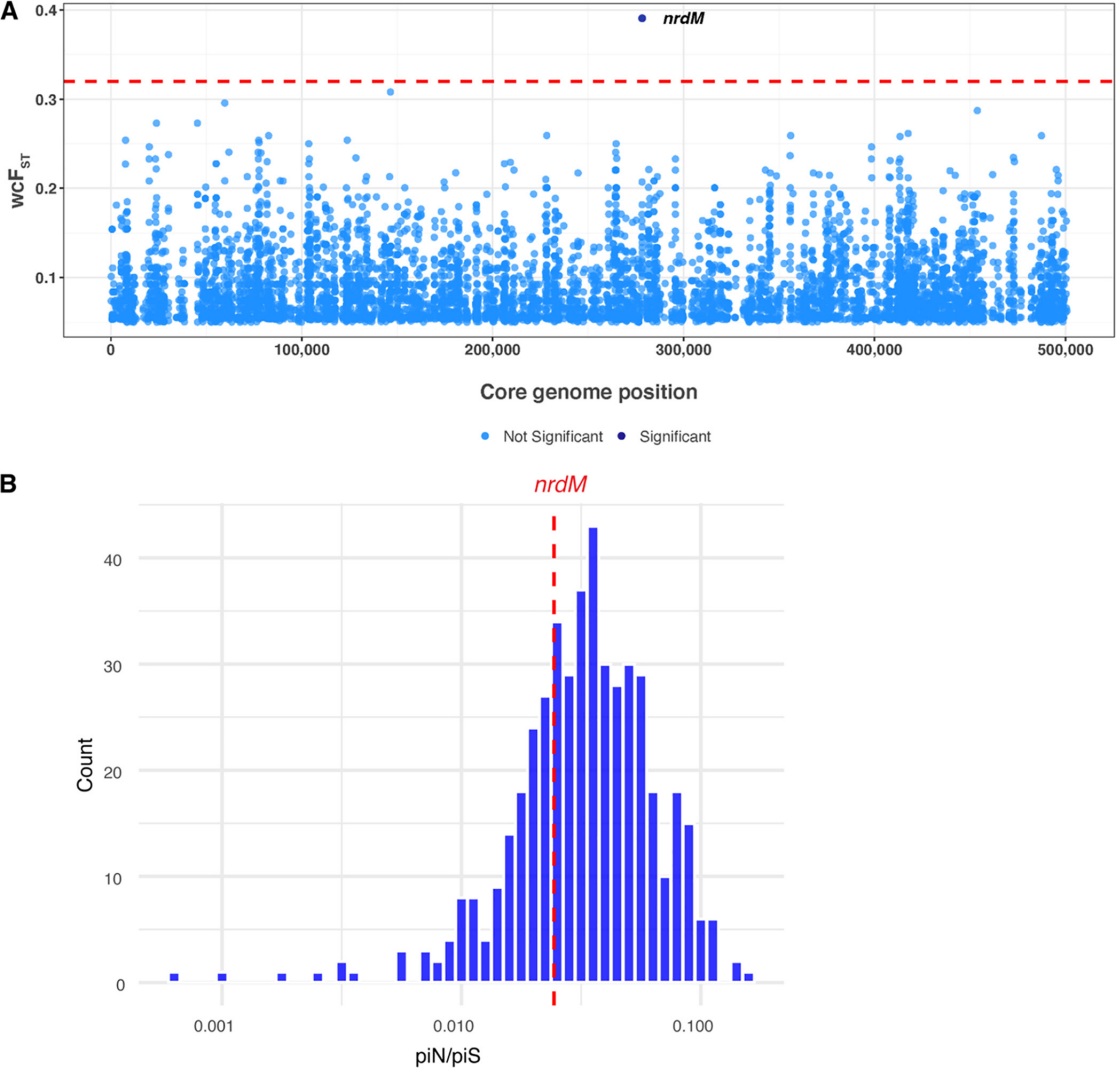
A single core genome variant was associated with invasiveness in S. oralis isolates. (A) Weir and Cockerham’s F_ST_ (wcF_ST_, abbreviated F_ST_) values were calculated for each core genome variant (*n* = 67,026) and plotted against the core genome position. The significance threshold (red dotted line) was estimated by taking the maximum F_ST_ value from 100 random permutations of phenotypes. Variants with nonsignificant F_ST_ values are shown in light blue, and a single variant with a significant F_ST_ value is shown in dark blue. (B) Pairwise piN/piS values were calculated and averaged for each core gene and plotted as a histogram. The average piN/piS value across all genes is 0.039, red dotted line represents the average piN/piS value for *nrdM* (0.024).

To identify core genome variants associated with invasiveness, we started with an F_ST_ outlier analysis, which delineated allele frequency differences between subpopulations and identified variants with extreme measures of differentiation. We defined subpopulations of our sample as being of “oral” or “blood” source and used vcflib (https://github.com/vcflib/vcflib) to calculate Weir and Cockerham’s F_ST_ (wcF_ST_, abbreviated to F_ST_) for biallelic SNPs (*n* = 67,026) in the core genome alignment (Fig. 4A). We identified a single SNP with a significant F_ST_ value, indicating significant allele frequency differences between oral and blood subpopulations of S. oralis (Fig. 4A). The SNP of interest lies within a gene originally annotated as a homolog of *nrdD*, an anaerobic ribonucleoside triphosphate reductase in many streptococci, involved in the synthesis of deoxyribonucleotides under anaerobic conditions (39). However, further inspection of the sequence revealed that the canonical *nrdD* gene was annotated separately in our S. oralis isolates and that the novel protein was shorter than *nrdD*. The novel protein did share some sequence features with *nrdD*, including an ATP cone domain, so we will refer to the novel locus as *nrdM*. Given the amount of recombination present in our sample (Fig. 3A), we validated the results of our F_ST_ outlier analysis using GWAS methods specifically designed for use with microbial genomes. We used two programs, treeWAS (40), which corrected for the presence of recombination, and BugWAS (41), which identified lineage effects and controls for population structure. Results from both tools replicated the results of our F_ST_ outlier analysis and returned a single variant that was significantly associated with the phenotype, the same SNP in *nrdM* (Fig. S2).

10.1128/msphere.00509-22.2 FIG S2 GWAS results confirmed the results of the F_ST_ outlier analysis. BugWAS (left) and TreeWAS (right) results, each showing a single significant variant associated with invasiveness. The same variant in *nrdM* was identified in the F_ST_ outlier analysis. Download FIG S2, PDF file, 0.1 MB. Copyright © 2022 Joyce et al. 2022 Joyce et al. https://creativecommons.org/licenses/by/4.0/ This content is distributed under the terms of the Creative Commons Attribution 4.0 International license.

The SNP of interest in *nrdM* was a synonymous mutation (I78I), and mapping the nucleotide alleles of the mutation (C233T) onto the core genome phylogeny showed interspersal of alleles on the tree, with some structure in subsp. *dentisani* and subsp. *tigurinus* (Fig. S3). Most interesting was the striking association between isolates from invasive infections and the *nrdM* allele. Out of 51 invasive isolates in our sample, 39 encoded the *nrdM* SNP associated with invasiveness (Fig. S3; Table S2). A multiple sequence alignment of NrdM from our sample of S. oralis strains revealed high protein sequence conservation (Fig. S4). To determine whether *nrdM* was conserved over other core genes, we calculated the average πN/πS (piN/piS) values for each core gene (*n* = 801). The distribution of gene-wise values of piN/piS indicated that most genes were evolving under relatively strong purifying selection, including *nrdM*, which had a piN/piS value slightly below the mean (Fig. 4B). This was not surprising given that recombination strengthens the efficiency of selection by enabling rapid removal of deleterious mutations (42).

10.1128/msphere.00509-22.3 FIG S3 Comparison between isolation source (oral commensal or blood/invasive infection) and *nrdM* allele (C or T) shown on mirrored core genome phylogenies. Tree tips connected by dotted lines have convergent genotype-phenotype combinations (*i.e.*, the *nrdM* allele associated with invasiveness in an isolate from an invasive infection). Download FIG S3, PDF file, 0.2 MB. Copyright © 2022 Joyce et al. 2022 Joyce et al. https://creativecommons.org/licenses/by/4.0/ This content is distributed under the terms of the Creative Commons Attribution 4.0 International license.

10.1128/msphere.00509-22.4 FIG S4 Multiple sequence alignment (MSA) of NrdM plotted next to the S. oralis core genome phylogeny where tips have been colored by the *nrdM* allele. The scale on top of the MSA represents the length of amino acids, and the position of the synonymous mutation of interest (I78I) is indicated with an arrow. Download FIG S4, PDF file, 0.1 MB. Copyright © 2022 Joyce et al. 2022 Joyce et al. https://creativecommons.org/licenses/by/4.0/ This content is distributed under the terms of the Creative Commons Attribution 4.0 International license.

### Selection on *nrdM* variant.

Given the strong association between the *nrdM* variant and invasiveness, we wondered if this SNP was under positive selection in our sample. One method for identifying positive selection was the identification of homoplastic mutations, i.e., mutations that arose more than once on the phylogeny, which we have used previously to screen for drug-resistant loci in Mycobacterium tuberculosis (43). We used TreeTime (44) to identify homoplastic mutations in our sample and found numerous homoplastic mutations. This was expected because intergenomic recombination can produce homoplasies by lateral transfer of sequence variants. The variant in *nrdM* arose 20 times on the phylogeny, which was in the 85^th^ percentile of mutation multiplicity (Fig. S5). This finding, along with gene-wise piN/piS values (Fig. 4B), suggested that selection pressures were similar at this locus to others in the genome. However, further analysis with a larger sample size could elucidate more subtle signs of selection.

10.1128/msphere.00509-22.5 FIG S5 Histogram of mutation multiplicity for all homoplastic mutations on the S. oralis core genome phylogeny. The red dotted line shows the 95^th^ percentile cutoff and the multiplicity of the *nrdM* SNP (n = 20) is indicated by the black arrow. Download FIG S5, PDF file, 0.1 MB. Copyright © 2022 Joyce et al. 2022 Joyce et al. https://creativecommons.org/licenses/by/4.0/ This content is distributed under the terms of the Creative Commons Attribution 4.0 International license.

### *nrdM* was conserved among VGS species.

**FIG 5:**
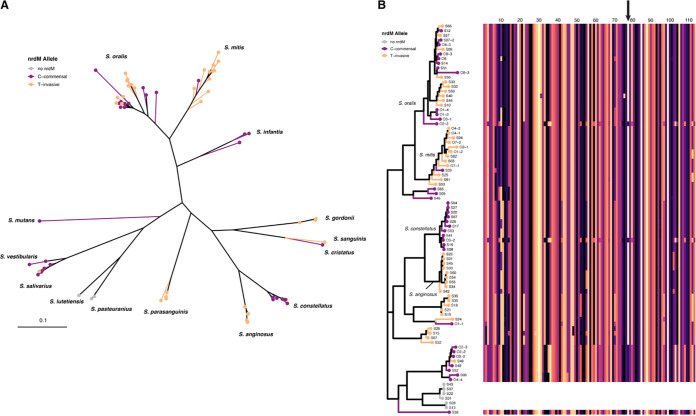
(A) Homologous *nrdM* genes identified in all newly sequenced VGS isolates with alleles from our variant of interest plotted on a phylogeny made from GyrB sequences. Only 2 species in our sample lacked *nrdM* (S. lutetiensis and S. pasteuranius), both of which were in the bovis group and are shown in gray. All other species contained *nrdM* and were conserved at the position of interest. (B) Multiple sequence alignment (MSA) of NrdM plotted next to the *gyrB* phylogeny (same tree as in [A]) where tips have been colored by the *nrdM* allele. The scale on top of the MSA represents the length of amino acids, and the position of the synonymous mutation of interest (I78I) is indicated with an arrow. Clade labels are shown for the most common species: S. oralis, S. mitis, S. anginosus, and S. constellatus.

To investigate the presence of *nrdM* homologs in other Streptococcus spp., we used BLAST to search annotated genes in all newly sequenced isolates and found *nrdM* to be present in all VGS species in our original sample, except for two bovis group species, S. lutetiensis and S. pasteuranius (Fig. 5A). Additionally, the position of the variant associated with invasiveness in S. oralis was conserved, and we were able to identify variation in *nrdM* alleles between different species. As in S. oralis, NrdM was highly conserved at the protein level across the VGS species in our sample, indicating the gene may have a conserved function across multiple species (Fig. 5B).

### Deletion of *nrdM* did not affect *in vitro* growth.

To investigate the impact the *nrdM* orphan allele has on growth in the two infective endocarditis strains S. oralis 1648 (SO48) and mitis group Streptococcus 1643 (SM43), we deleted *nrdM*, locus tags MP387_03665, and FD735_06230, respectively, via homologous recombination (Table 1). SO48 possessed the *nrdM*-C (“C”, commensal) allele, and SM43 possessed the *nrdM*-I (“I”, invasive) allele. Knockout mutants were confirmed via Sanger sequencing. No difference in growth was observed for knockout mutants compared to wild-type in Todd-Hewitt broth (THB) (Fig. S6). Further, *nrdM* allele swaps from SO48 and SM43 were placed back into the genome of SO48Δ*nrdM* and SM43Δ*nrdM* to generate Δ*nrdM*::*nrdM*-I or Δ*nrdM*::*nrdM*-C strains in each strain background (Table 1). Because the presence of human serum has been shown to impact mitis group streptococci physiology (45, 46), we assessed the impact of *nrdM* during growth with human serum by performing growth curves of allele replacement strains in chemically defined medium supplemented with 5% vol/vol human serum (Fig. S6). No difference in growth profile was observed between alleles in either strain background. Thus, the *nrdM* locus does not impact growth in rich laboratory medium or chemically defined medium supplemented with 5% vol/vol human serum *in vitro*.

**TABLE 1:** Strains used in this study

Organism	Strain	Description	Reference
Mitis group streptococci	1643 (SM43)	Wild-type infective endocarditis isolate	19
	SM43ΔNrdM	SM43 with clean deletion of *nrdM*, FD735_06230	This work
	SM43:NrdM-I	SM43 with SM43 *nrdM* allelic replacement into SM43Δ*nrdM*	This work
	SM43:NrdM-C	SM43 with SO48 *nrdM* allelic replacement into SM43Δ*nrdM*	This work
S. oralis	1648 (SO48)	Wild-type infective endocarditis isolate	19
	SO48ΔNrdM	SO48 with clean deletion of *nrdM*, MP387_03665	This work
	SO48:NrdM-I	SM43 *nrdM* allelic replacement into SO48Δ*nrdM*	This work
	SO48:NrdM-C	SO48 *nrdM* allelic replacement into SO48Δ*nrdM*	This work

10.1128/msphere.00509-22.6 FIG S6 Growth curves of mutant strains. Growth curves of wild-type and Δ*nrdM* strains of (A) SM43 and (B) SO48 performed in Todd-Hewitt Broth, in biological duplicate. OD600 readings were performed every hour for 8 hrs. (The mean and SD are indicated. Growth of NrdM-I and NrdM-C alleles in [C]) SM43 and (D) SO48 strain backgrounds when grown in streptococcal defined medium supplemented 5% v/v human serum. Serum growth was performed in biological triplicate and OD600 readings were taken at 0, 3, 6, and 24 hrs. The mean and SEM are indicated. Download FIG S6, PDF file, 0.1 MB. Copyright © 2022 Joyce et al. 2022 Joyce et al. https://creativecommons.org/licenses/by/4.0/ This content is distributed under the terms of the Creative Commons Attribution 4.0 International license.

### *nrdM* had no impact on growth in a simulated infective endocarditis vegetation model.

**FIG 6:**
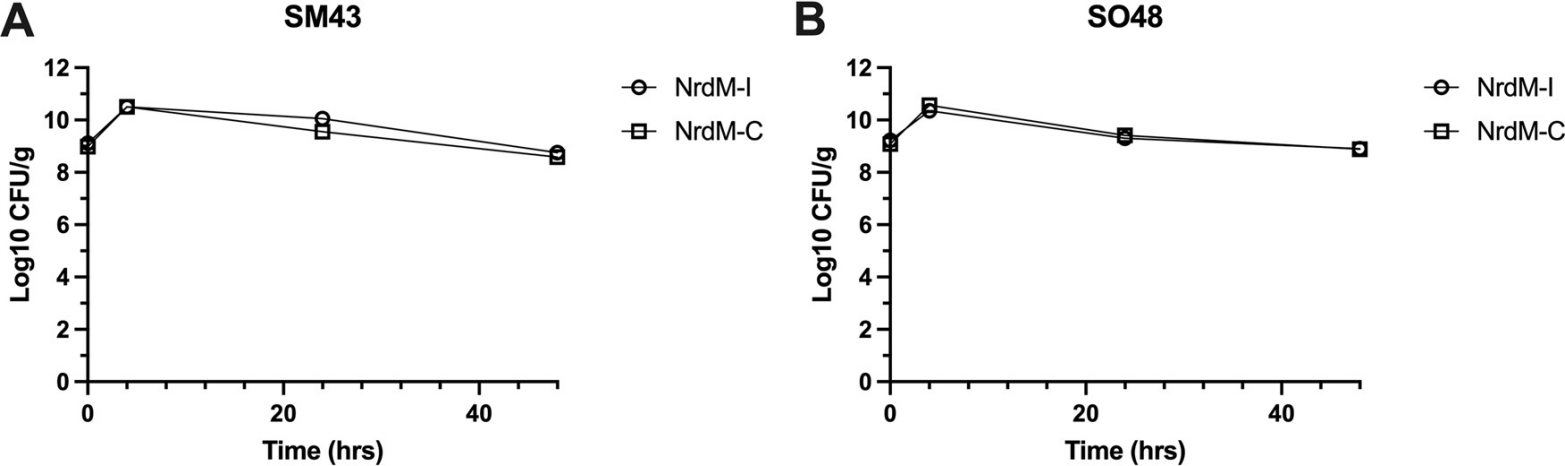
Simulated infective endocarditis vegetation model of *nrdM* allele swapped strains. Survival of either (A) SM43 or (B) SO48 harboring either the infectious *nrdM*-I allele or the commensal *nrdM*-C allele during vegetation growth. Biological triplicates were performed for each strain with four technical replicates per time point. The mean and SEM are indicated. No significant difference was observed under these conditions. Two-way ANOVA with Sidak’s multiple-comparison test.

Finally, the allele-swapped strains were investigated using the pharmacological *in vitro* simulated infective endocarditis vegetation model (SIEVM). We reasoned that if *nrdM*-I conferred enhanced fitness in simulated endocardial vegetations, we would observe significantly different vegetation CFU for SO48Δ*nrdM*::*nrdM*-I versus SO48Δ*nrdM*::*nrdM*-C. The SM43 strain was similarly tested, to assess the effect of *nrdM* allele swapping in a closely related but different (i.e., non-*oralis*) genetic background. Strains were inoculated into vegetation clots at ~1 ×10^8^ CFU and incubated in the chemostat model with Mueller-Hinton broth (MHB) in parallel for 48 h (see Materials and Methods). At 4, 24, and 48 h postinoculation, 4 clots from each model were removed for CFU enumeration (Fig. 6). No significant difference was observed during growth in the SIEVM between *nrdM* allele swap in either strain background. Ultimately, these data and the growth curve data together showed that *nrdM* was not essential under these *in vitro* conditions, and no phenotype could be assigned to either of the *nrdM* alleles.

## DISCUSSION

The viridans group streptococci (VGS) are a large collection of closely related Streptococcus spp. that inhabit the oral cavity and gastrointestinal and genitourinary tracts of humans as commensals but can invade other tissues to cause severe diseases, such as bacteremia and infective endocarditis (1). Our understanding of the pathogenesis of VGS disease is limited by a lack of knowledge surrounding the genetic and environmental conditions that facilitate a switch from commensalism to the pathogen. Additionally, clinical detection and identification of bacterial infections are critical for patient care and recovery, especially in neutropenic and immunocompromised patients. A major etiological agent of disease in this patient demographic are the VGS, especially S. mitis and S. oralis. Yet, clinical methods of identification are still largely inaccurate, and even newer methods like MALDI-TOF MS struggle to differentiate between closely related species, such as S. oralis and S. mitis (7). In this study, we collected and whole-genome-sequenced a variety of invasive and noninvasive VGS isolates, compared bioinformatic methods for delineating closely related species, and identified an SNP that may predispose certain isolates of S. oralis to invasive disease.

### Complex population structure of S. oralis.

We characterized the population structure of 108 S. oralis isolated from healthy oral microbiota and blood and found high levels of diversity in the core (Fig. 2B and C) and accessory genomes (Fig. 2A and D). We found that, using our methods, previously described subspecies of S. oralis corresponded to subclades that we identified in a phylogeny inferred from core genome sequences (Table S2). We also identified subsp. *oralis* as being the least diverse of the three subspecies, with respect to both gene content and sequence variation in the core genome (Fig. 2). We asserted that recombination was likely the primary mechanism for generating and maintaining diversity in this species because 99.9% of the S. oralis core genome had been affected by recombination (Fig. 3). The rarity of a given recombinant fragment in our sample also indicated that S. oralis isolates were likely participating in HGT with diverse species, which was made possible because they inhabited complex communities. We observed a mean piN/piS value across S. oralis core genes of 0.039, which is an order of magnitude lower than comparator species, such as S. aureus for which values of 0.32 (47) and 0.55 (48) have been reported and lung-colonizing Pseudomonas aeruginosa from cystic fibrosis patients that have a mean of 0.14. (49). This indicates that S. oralis core genes are under strong purifying selection. We recently identified a similar phenomenon among environmental isolates of Mycobacterium abscessus that are also highly recombinogenic and manifest large amounts of synonymous diversity in their core genomes (50). This suggests that like M. abscessus, S. oralis inhabits environments alongside diverse microbial species where high rates of recombination enable the ready acquisition of novel genetic material and rapid removal of deleterious mutations.

### Switching from commensal to pathogenic.

Our identification of extremely high recombination rates within S. oralis points to the unique genomic features characterizing this VGS species. High rates of recombination allow the species to adapt to fluctuating environments encountered within the human body, and potentially enable the invasion of new niches, including pathogenic niches. We used three different GWAS methods that reproducibly identified a strong association between a synonymous SNP within an undescribed protein, NrdM, and invasive disease (Fig. 4, Fig. S2). The association was robust across diverse isolates of all three subspecies (Fig. S3). We also found that *nrdM* was conserved among S. oralis isolates (Fig. S4) and was one of only ~440 genes conserved across all VGS species in our sample, indicating that it was likely to play an important, and perhaps a similar, role across species. Utilizing *in vitro* growth in the presence of human serum and a simulated infective endocarditis vegetation model, we compared *nrdM* knockout strains and allele-swapped invasive and commensal isolate alleles in two strain backgrounds. No phenotype was observed under the tested conditions, suggesting that if NrdM is contributing to invasive disease, it requires different conditions (possibly specific to the *in vivo* environment) for a potential phenotype to be observed. NrdD, which shares the same ATPase cone domain as NrdM, is required for anaerobic growth in S. sanguinis (39), and, notably, disruption of this gene results in attenuated virulence (51). Long thought to have neutral fitness effects, synonymous mutations are increasingly recognized as having significant effects on bacterial fitness, for example by impacting gene expression and protein folding (52–56). We believe that further study of this synonymous variant could reveal fitness effects and that adaptation within *nrdM* may affect a multitude of phenotypes that aid in the transition from the oral cavity to the bloodstream, although further investigation is necessary.

A major hurdle to successful genomic analyses in VGS is the correct classification of the different species. Incorrect species identification may have contributed to the lack of clear results from studies looking at the genetic determinants of virulence in VGS species. One such study by Rasmussen et al. (57) used a sample of both S. mitis and S. oralis to search for known virulence factors, which they found at various frequencies, indicating that genetic differences may be responsible for variability in virulence among strains. Similar to our sample of S. oralis isolates (Fig. 2B), studies of the closely related VGS S. sanguinis and S. gordonii were unable to identify phylogenetic patterns based on invasive disease (58, 59). These studies, however, were unable to identify specific genomic variants associated with invasiveness. What our work and the work of others indicate is that virulence properties differ between VGS species, despite being so closely related and often causing similar diseases. This is further illustrated by the fact that although *nrdM* is conserved in S. mitis, the association between the invasive allele identified in S. oralis and pathogenicity in S. mitis is not significant (unpublished data). Going forward, analyses using larger samples of well-defined, individual species will provide better resolution for identifying the genetic determinants of virulence in the VGS.

## MATERIALS AND METHODS

### Collection of clinical isolates.

Clinical strains obtained from routine blood cultures and identified as viridans group streptococci were stored in the microbiology laboratory at each hospital (Methodist Health System [MHS] and University of Mississippi Medical Center [UMMC]). Respective site investigators reviewed patient clinical information to confirm invasive infection (i.e., bacteremia or endocarditis), then deidentified specimens and provided blinded isolates to the University of Texas at Dallas (UTD) laboratory for study. Specimens were not included for further analysis if the isolate was deemed a contaminant and did not require antibiotic therapy as determined by the treating physician. Site investigators obtained approval from respective Institutional Review Boards (MHS UTD IRB 18–121 and UMMC 2018-0068).

### Processing of clinical isolates.

Clinical isolates were struck on Mitis-Salivarius agar (MSA) (BD Bacto) and incubated overnight at 37°C and 5% CO_2_. MSA plates were observed for homogenous colony morphology, and a single colony was inoculated into 10 mL THB for overnight growth at 37°C and 5% CO_2_. If more than one colony morphology was identified on MSA plates, broth cultures were made from each colony morphology. Overnight cultures were stored at −80°C in 25% glycerol. The remaining culture volume was pelleted at 4,280 × *g* in a Sorvall RC6+ floor centrifuge and genomic DNA was extracted using Qiagen DNeasy blood and tissue kit per manufacturer protocols, with minor modifications as described in (6).

### Collection of oral swab samples.

Oral swabs were obtained from healthy adult volunteers at the UTD campus (UTD IRB 17-170). The exclusion criteria applied included a history of bacteremia or endocarditis, recent antibiotic exposure (prior 30 days), history of periodontal disease, and personal or family history of being immunocompromised. No participants were excluded based on these criteria. The volunteer was asked to rinse their mouth with sterile saline, and then self-swab their teeth and tongue with a sterile swab (Puritan). Swabs were stored at 4°C until processing.

### Processing of oral swabs.

Oral swabs were processed as described above. Briefly, swabs were struck onto MSA plates and grown overnight. After incubation, the MSA plates were observed and colony morphologies consistent with Streptococcus mitis and S. oralis were selected for overnight growth in THB. In addition, approximately 3 random colonies of different morphologies were also selected for overnight growth. Cultures were processed as described above.

### 16S rRNA sequence analysis and GyrB typing.

PCRs were performed using *Taq* polymerase (New England Biolabs) with primer sequences in Table S3. 16S rRNA genes were amplified using universal primers 8F and 1492R (60). The DNA Gyrase B gene (*gyrB*) was amplified using previously reported primers (Table S3). PCRs were analyzed by agarose gel electrophoresis and purified using the GeneJET PCR purification kit (Thermo Fisher) per the manufacturer's protocols. Products were sequenced at the Massachusetts General Hospital DNA Core. 16S rRNA sequences were trimmed using Geneious R11 (https://www.geneious.com) allowing a maximum of 10 low-quality bases and 6 ambiguities. Trimmed sequences were used as queries for NCBI BLASTN against the 16S rRNA sequences (bacteria and archaea) database and species were assigned only when the forward and reverse sequencing reactions had the same top BLASTN result (Table S1). GyrB nucleotide sequences were translated, and amino acid sequences pairwise aligned to Streptococcus mitis ATCC 49456 GyrB (locus tag SM12261_0755). Amino acid variations were identified using the method of Galloway-Peña et al. (11).

10.1128/msphere.00509-22.9 TABLE S3 Primers used in this study. Download Table S3, PDF file, 0.1 MB. Copyright © 2022 Joyce et al. 2022 Joyce et al. https://creativecommons.org/licenses/by/4.0/ This content is distributed under the terms of the Creative Commons Attribution 4.0 International license.

### Ilumina sequencing.

Sequencing was performed at the University of Texas at Dallas Genome Core using Illumina Nextseq 500 platform with a midoutput 300 cycle of 2 × 75 bp paired-end reads for clinical isolates S1-S31 or 2 × 150 bp paired-end reads for all other isolates.

### Genome assembly and annotation.

Using the raw sequencing data from newly sequenced clinical and oral isolates (Table S1), species identification was additionally performed using Kraken2 (20). Raw data were quality-checked and trimmed using FastQC v0.11.8 (61) and TrimGalore v0.6.4, (http://www.bioinformatics.babraham.ac.uk/projects/trim_galore), respectively. Contigs were assembled using SPAdes v3.13.0 with default parameters (62). Assemblies were checked for quality using Quast v5.0.2 (63) filtering out contigs shorter than 500 bp or with coverage lower than 5×, as well as confirming all assemblies had an *N*_50_ > 50,000 bp. Contigs were annotated using Prokka v1.13.3 (64) before a pangenome analysis was performed with Roary v3.12.0 using a blastp identity threshold of 75% (31). Using a nucleotide sequence alignment of GyrB (as clustered by Roary) a phylogenetic tree was made using FastTree v2.1.9 (65) and visualized in R with ggtree (66). GyrB typing was confirmed using these sequences with a custom script (code available at https://github.com/myoungblom/VGS_GWAS.git) assigning species based on the scheme outlined by Galloway-Peña et al. (11). *nrdM* sequences from all newly sequenced VGS isolates were identified with BLASTP using the S. oralis NrdM amino acid sequence as the query sequence.

### S. oralis genome collection for GWAS analyses.

From our sample of clinical and oral isolates, S. oralis made up the largest part of our sample. Thus, we decided to proceed with analyses of just this species. To create a data set large enough for a powerful genome-wide association study (GWAS) we identified all S. oralis and S. mitis isolates from NCBI (NCBI Sequence Read Archive (SRA) and assembly databases accessed June 2019) with the proper metadata indicating they were isolated from the mouth (e.g oral cavity, dental plaque, dental biofilm, etc.) or blood (e.g., infective endocarditis, bloodstream infection, blood, etc.). We assumed isolates uploaded to NCBI with various “oral” sources were all commensal and all those from “blood” were from an invasive infection (Table S2). We chose to start with both S. oralis and S. mitis because these species were closely related and they were often mistaken for each other and uploaded to NCBI under the wrong species (Table S2), as has previously been reported (10). We pulled out the true S. oralis isolates using Kraken and GyrB typing as described above. Samples for which raw sequence data were available were assembled as described above and then annotated with the remainder of assemblies downloaded from NCBI (Table S2). Because of the time of data collection, three of the assemblies used in these data sets have been suppressed (Table S2). We then performed a pangenome analysis on the S. oralis sample as described above (using a BLASTP identity threshold of 95% and Prank to align core genes) and a phylogenetic tree was inferred from the resulting core genome alignment using RAxML v8.2.3 (67) and visualized in R with ggtree (66).

### Recombination analyses.

We identified recombinant fragments in the S. oralis core genome using Gubbins v2.4.1 with default parameters (35). Recombinant fragments were visualized alongside the core genome phylogeny using Phandango (68). We used ClonalFrameML v1.11 (36) with default parameters to estimate *r/m*.

### Population genetics statistics.

Pairwise average nucleotide identity (ANI) values of S. oralis core genome sequences were calculated with OrthoANI (69). piN/piS values were calculated for all pairwise combinations for each S. oralis core gene were calculated using Egglib (70), and then the average piN/piS value for each gene was calculated.

### Rarefaction and accumulation plots.

Rarefaction and accumulation curves for the S. oralis subspecies were calculated from Roary gene presence-absence files. Briefly, separate pangenome analyses were performed as described above for each subspecies and then each data set was iteratively subsampled to the size of the smallest data set (*n* = 25) and the median number of core and total genes was plotted from all iterations.

### GWAS.

We first queried for genetic associations with the ‘invasive’ phenotype in our data set by identifying accessory gene content significantly associated with invasiveness using Scoary v1.16.6 (38). We then performed a preliminary GWAS of core genome variants using an F_ST_ outlier analysis. Briefly, a VCF file containing all core genome variants was made using SnpSites v2.0.3 (71) and reformatted using a custom script (code available https://github.com/myoungblom/VGS_GWAS.git). We then calculated Weir and Cockerham’s F_ST_ for biallelic SNPs using vcflib (https://github.com/vcflib/vcflib). Using a custom script (code available at https://github.com/myoungblom/VGS_GWAS.git), we permuted the phenotypes in this analysis 100× and used the maximum F_ST_ value observed in the null distribution as a cutoff to identify significant F_ST_ outliers. To validate the results of our F_ST_ outlier analysis, we also used two GWAS programs designed specifically for use with microbial genomes. These included treeWAS (40), which corrected for the presence of recombination, and BugWAS (41), which identified lineage effects and controls for population structure. TreeWAS was run using the recombination-adjusted phylogenetic tree made with Gubbins (see above) using 10× the number of SNPs in the core genome for the parameter “n.snps.sim.” BugWAS was run using default parameters.

### Homoplasy analysis.

Homoplasy analysis was performed using TreeTime v0.9.0-b.2 (44) with default parameters.

### Deletion of *nrdM*.

Knockout constructs of *nrdM* in SM43 (locus ID FD735_06230) and SO48 (locus ID MP387_03665) were generated as previously described (45). Briefly, linear constructs were generated by amplifying ~2 kb regions upstream and downstream of *nrdM* using Phusion polymerase (Thermo Fisher) using primers in Table S3. SOEing PCR was used to stitch fragments together and the amplified product was assessed via agarose gel electrophoresis. Gel extraction was performed using the QIAQuick Gel Extraction kit (Qiagen). Linear constructs were transformed by natural transformation as described in (45). Transformation plates were incubated overnight, and putative transformant colonies were screened via PCR for the *nrdM* deletion.

### *nrdM* allele swaps.

Allele swap strains were generated using the same strategy as the deletion, except the linear construct contained either the *nrdM*-I or *nrdM*-C allele coupled with the flanking regions for the respective strain. The linear construct was transformed into SM43Δ*nrdM* and SO48Δ*nrdM.* The allele swap region in transformants was amplified using primers in Table S3. Products were sequenced for validation of the allele swap (Massachusetts General Hospital DNA Core).

### Growth curves.

Growth curves in THB were performed in biological duplicate. Wild-type and Δ*nrdM* strains were grown overnight as described and then diluted to an optical density at 600 nm (OD_600_) of 0.05 in approximately 12 mL THB. The OD_600_ was monitored every hour using a Thermo Scientific Genesys 30 spectrophotometer. For growth curves in the presence of human serum, biological triplicate overnight cultures were grown in streptococcal defined medium (45, 46, 72) and diluted to an OD_600_ of 0.1 in a defined medium supplemented with 5% vol/vol human serum (Sigma-Aldrich). The OD_600_ was monitored at 3, 6, and 24 h as described above.

### Simulated infective endocarditis vegetation model.

Strains were inoculated from freezer stocks into 5 mL Mueller-Hinton broth (MHB) (BD Bacto) and incubated overnight at 37°C and 5% CO_2._ Next, 1 mL was expanded into 100 mL prewarmed MHB and incubated overnight as described above. The SIEVM was set up and performed as previously described (19) using human blood products from the American Red Cross (UTD IRB 19MR0160). Briefly, 500 μL of pooled human cryoprecipitate (American Red Cross), 50 μL ~2 TIU/mL aprotinin (Sigma-Aldrich), ~100,000 human platelets (American Red Cross), and 10^8^ CFU/g bacteria were combined in a sterile Eppendorf tube and vortexed. A sterile monofilament line was positioned before 100 μL of ~2 KU/mL high-activity bovine thrombin (Sigma-Aldrich) was added to congeal the vegetation. Vegetations were placed into the glass apparatus in a 37°C water bath, and MHB was pumped through the model at a precalibrated rate of 0.4 mL/min. Four vegetations were removed at designated time points for each strain, weighed, removed from the monofilament line, and placed in 1.25% trypsin solution (Sigma-Aldrich) in sterile screw cap microcentrifuge tubes (Fisher Scientific) with ~5 to 8 2.7 mm glass beads (BioSpec). Clots were homogenized for 10 to 15 min horizontally on a vortex before serial dilution and plating on THB agar plates for enumeration. CFU/g was calculated by multiplying the observed CFU/mL by the net weight of the vegetation. SIEVM was performed in biological triplicate for each strain, with four vegetations per time point per strain.

### S. oralis 1648 hybrid genome assembly.

Pacific Biosciences single molecule real-time (SMRT) sequencing was performed by the Johns Hopkins Genome Core. The SO48 whole genome was assembled using the Unicyler assembly pipeline (73) combining SMRT long reads generated in this study and Illumina reads previously generated for SO48 (accession number PRJNA354070) (74).

### Data availability.

The SO48 whole-genome sequence generated in this study has been deposited in GenBank under the accession number CP094226. Genome constructs and Illumina and SMRT sequence reads generated in this study have been deposited in the Sequence Read Archive under the BioProject accession number PRJNA817585, see Table S1.
