# Erratum for Joyce et al., “Comparative Genomics of *Streptococcus oralis* Identifies Large Scale Homologous Recombination and a Genetic Variant Associated with Infection”

**DOI:** 10.1128/msphere.00266-24

**Published:** 2024-04-23

**Authors:** Luke R. Joyce, Madison A. Youngblom, Harshini Cormaty, Evelyn Gartstein, Katie E. Barber, Ronda L. Akins, Caitlin Pepperell, Kelli L. Palmer

## ERRATUM

Volume 7, no. 6, e00509-22, 2022, https://doi.org/10.1128/msphere.00509-22. Page 8, paragraph starting “The SNP of interest in *nrdM*,” first sentence: “(C233T)” should read “(C234T).”

