# Supplementary material for: Comparative Genomics of Streptococcus oralis Identifies Large Scale Homologous Recombination and a Genetic Variant Associated with Infection
Source: mSphere. 2022 Nov 2;7(6):e00509-22. doi: 10.1128/msphere.00509-22 (PMC9769543; doi:10.1128/msphere.00509-22)
Supplement: FIG S1 [file msphere.00509-22-s0004.pdf]

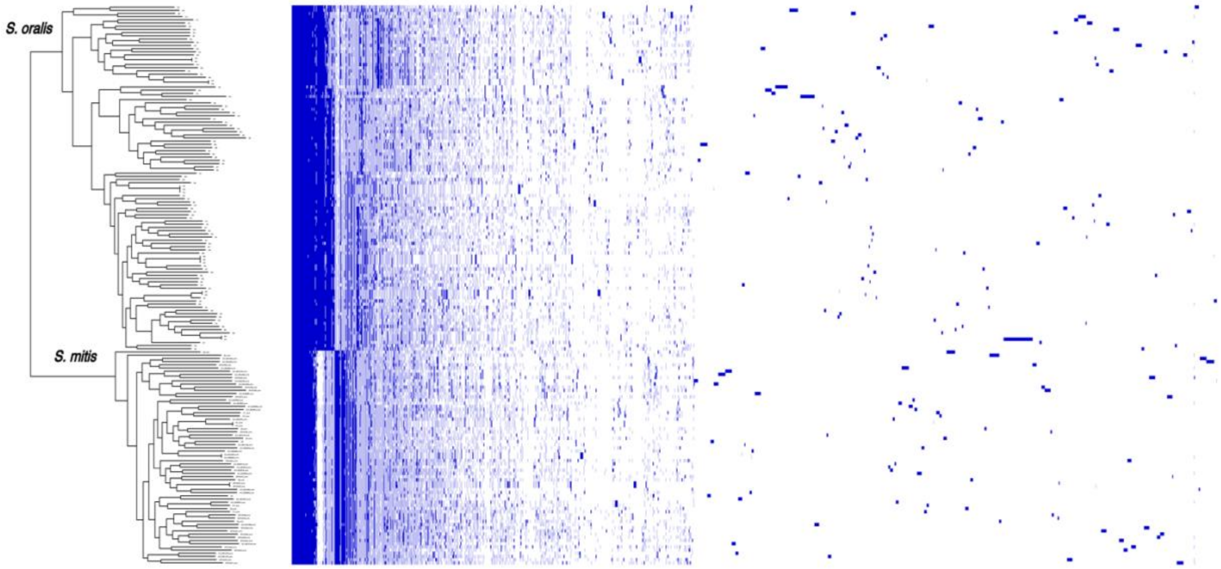


**Figure S1:** Core genome phylogeny of all *S. oralis* and *S. mitis* isolates (from newly sequenced samples and from NCBI) alongside a matrix of gene content. Clear delineation between *S. oralis* and *S. mitis* can be seen both in the core genome sequence as well as in core and accessory gene content differences between the species. Pangenome analysis performed using Roary and visualization with Phandango.
