# Supplementary material for: Comparative Genomics of Streptococcus oralis Identifies Large Scale Homologous Recombination and a Genetic Variant Associated with Infection
Source: mSphere. 2022 Nov 2;7(6):e00509-22. doi: 10.1128/msphere.00509-22 (PMC9769543; doi:10.1128/msphere.00509-22)
Supplement: FIG S2 [file msphere.00509-22-s0005.pdf]

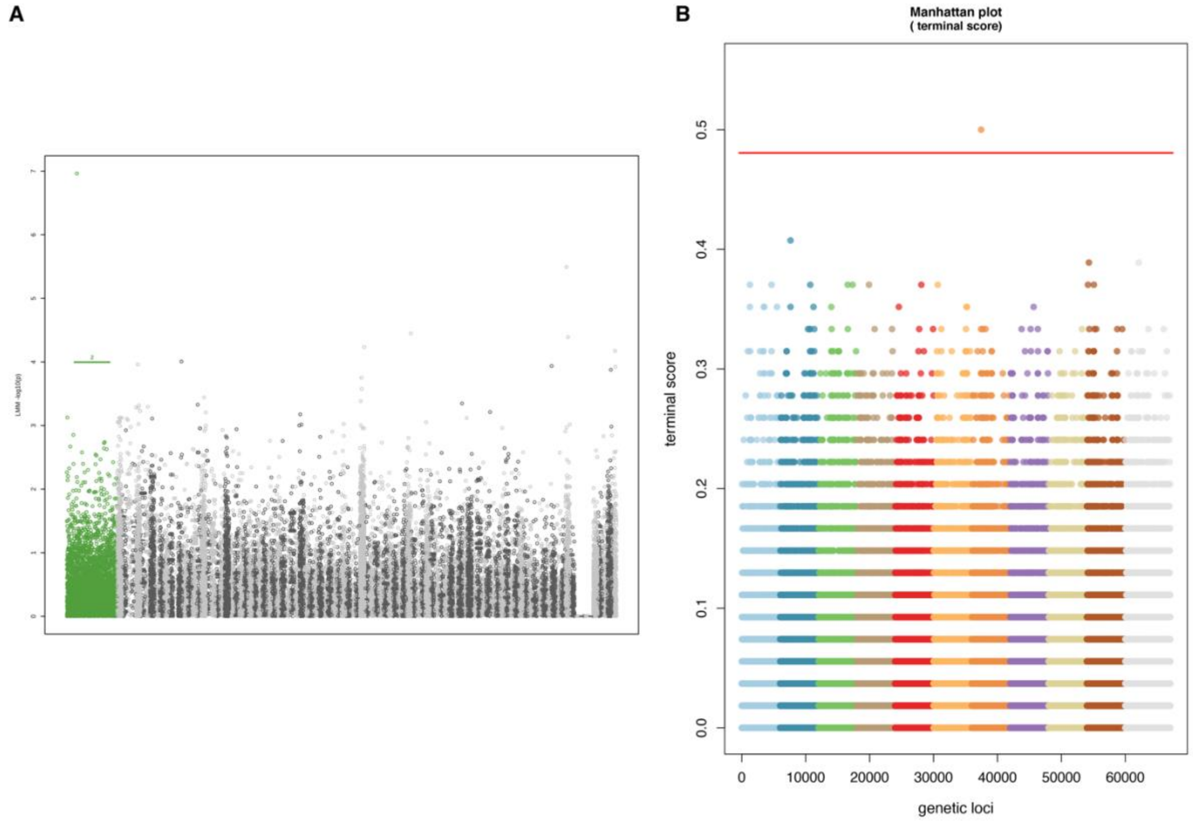

Figure S2: GWAS results confirming results of F_{ST} outlier analysis. BugWAS (left) and TreeWAS (right) results each showing a single significant variant associated with invasiveness: the same variant in *nrdM* identified in the F_{ST} outlier analysis.
