# Supplementary material for: Comparative Genomics of Streptococcus oralis Identifies Large Scale Homologous Recombination and a Genetic Variant Associated with Infection
Source: mSphere. 2022 Nov 2;7(6):e00509-22. doi: 10.1128/msphere.00509-22 (PMC9769543; doi:10.1128/msphere.00509-22)
Supplement: FIG S3 [file msphere.00509-22-s0006.pdf]

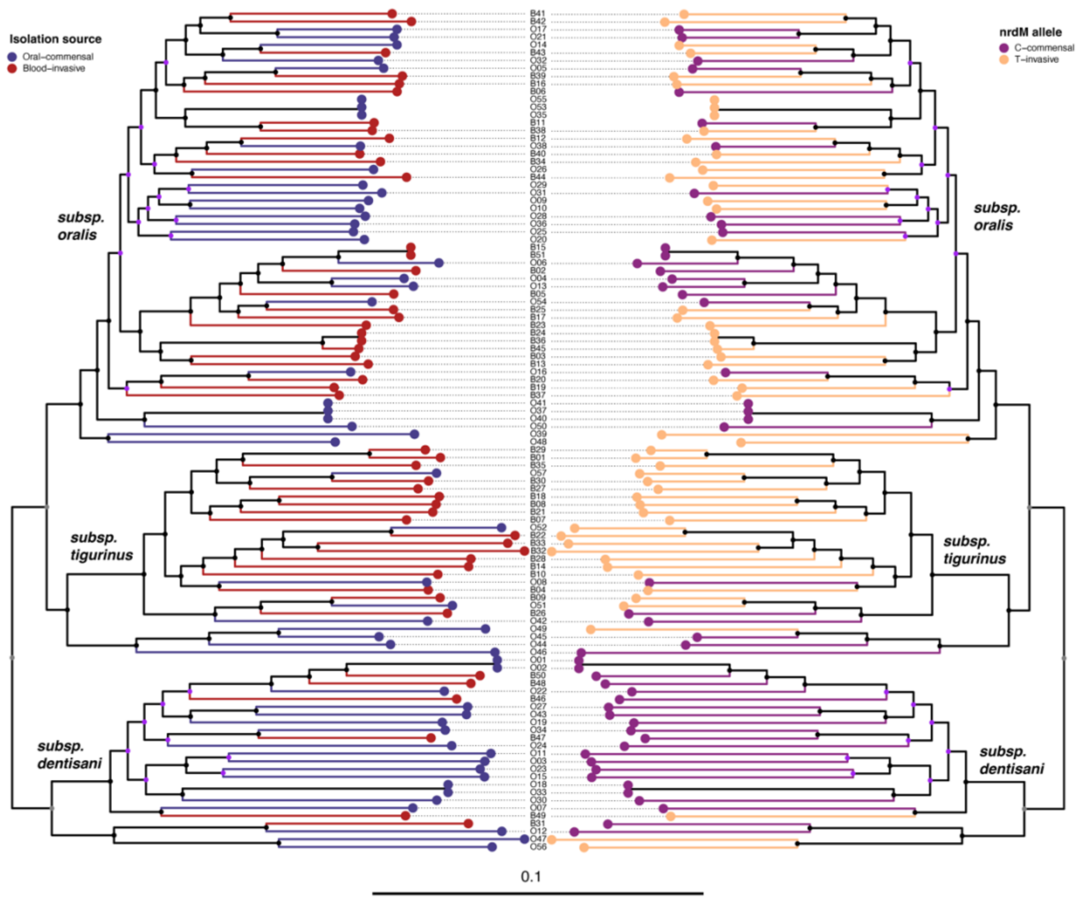

Figure S3: Comparison between isolation source (oral commensal or blood/invasive infection) and *nrdM* allele (C or T) shown on mirrored core genome phylogenies. Tree tips connected by dotted lines have convergent genotype-phenotype combinations (*i.e.* the *nrdM* allele associated with invasiveness in an isolate from an invasive infection).
