# Supplementary material for: Comparative Genomics of Streptococcus oralis Identifies Large Scale Homologous Recombination and a Genetic Variant Associated with Infection
Source: mSphere. 2022 Nov 2;7(6):e00509-22. doi: 10.1128/msphere.00509-22 (PMC9769543; doi:10.1128/msphere.00509-22)
Supplement: FIG S4 [file msphere.00509-22-s0007.pdf]

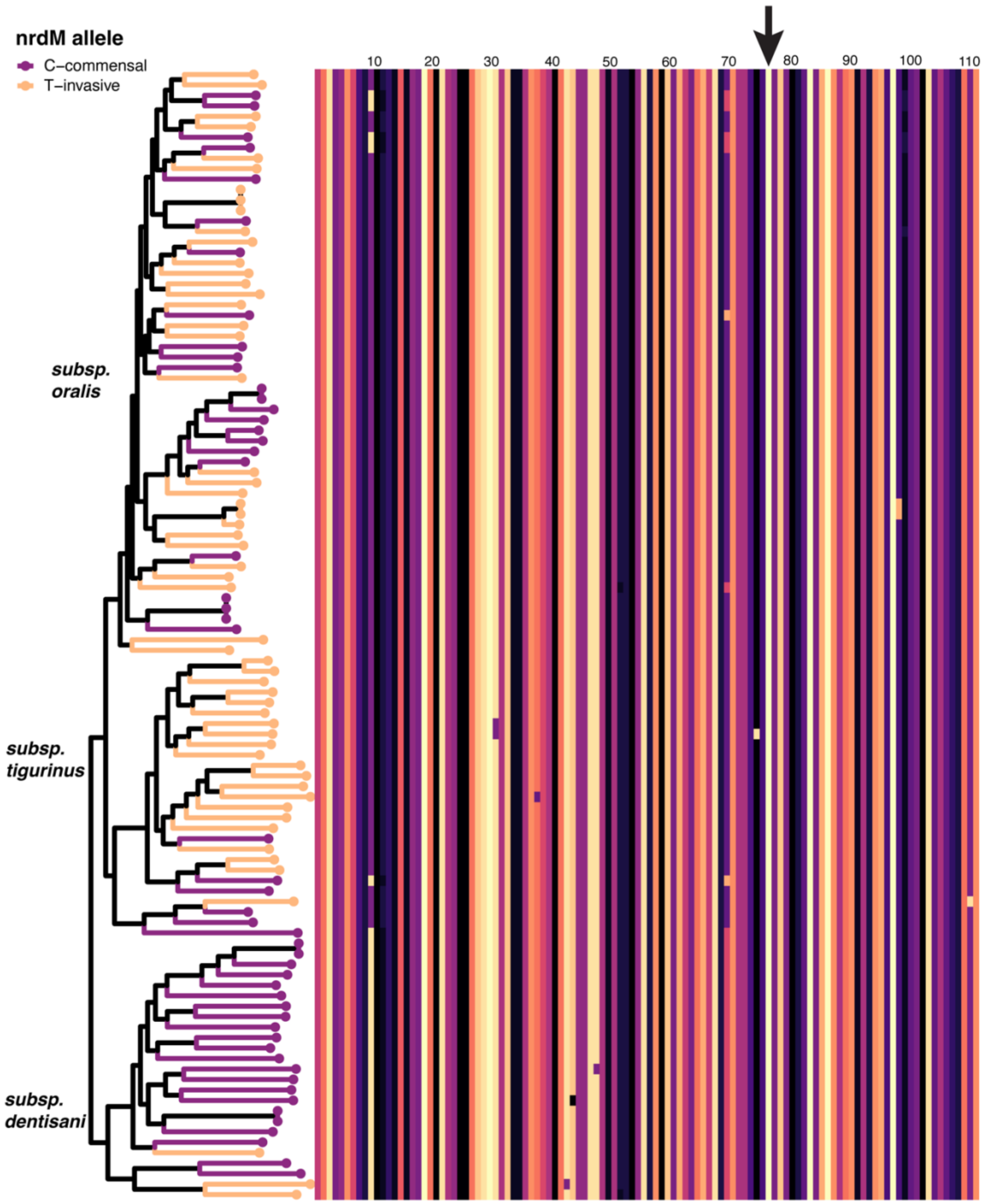

Figure S4: Multiple sequence alignment (MSA) of NrdM plotted next to the *S. oralis* core genome phylogeny where tips have been colored by *nrdM* allele. Scale on top of the MSA represents length in amino acids, the position of the synonymous mutation of interest (I78I) indicated with an arrow.
