# Supplementary material for: Comparative Genomics of Streptococcus oralis Identifies Large Scale Homologous Recombination and a Genetic Variant Associated with Infection
Source: mSphere. 2022 Nov 2;7(6):e00509-22. doi: 10.1128/msphere.00509-22 (PMC9769543; doi:10.1128/msphere.00509-22)
Supplement: FIG S5 [file msphere.00509-22-s0008.pdf]

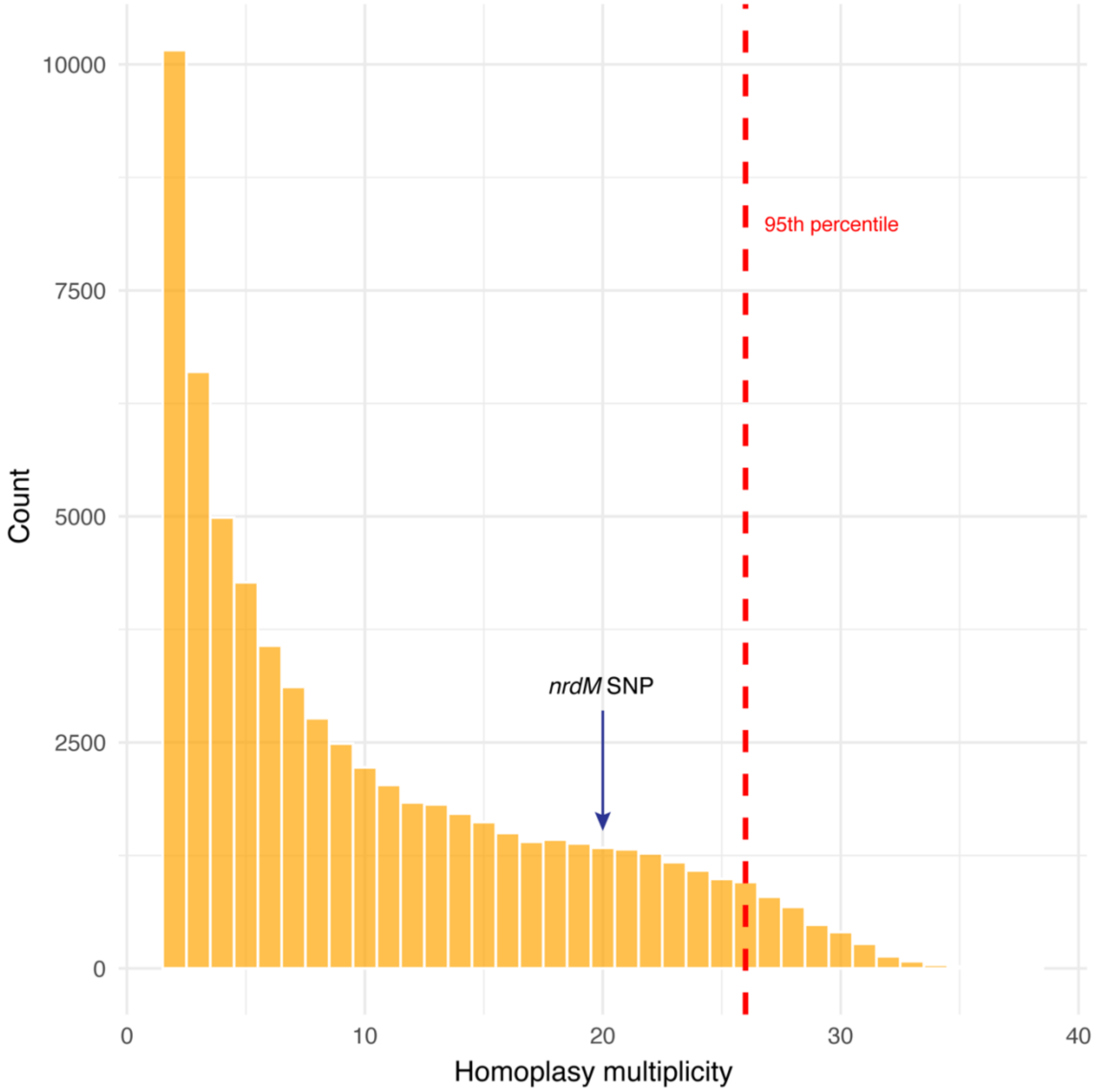

Figure S5: Histogram of mutation multiplicity for all homoplastic mutations on the *S. oralis* core genome phylogeny. Red dotted line shows 95th percentile cutoff and the multiplicity of the *nrdM* SNP (20) indicated by the black arrow.
