# Supplementary material for: Comparative Genomics of Streptococcus oralis Identifies Large Scale Homologous Recombination and a Genetic Variant Associated with Infection
Source: mSphere. 2022 Nov 2;7(6):e00509-22. doi: 10.1128/msphere.00509-22 (PMC9769543; doi:10.1128/msphere.00509-22)
Supplement: FIG S6 [file msphere.00509-22-s0009.pdf]

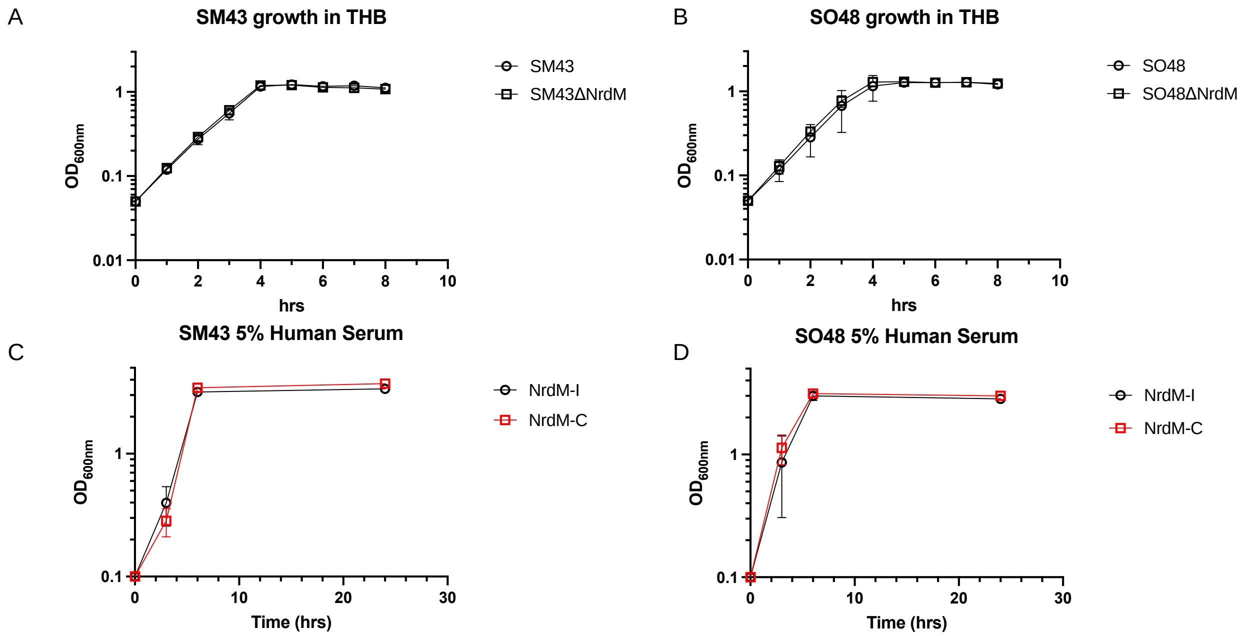


**Figure S6: Growth curves of mutant strains**. Growth curves of wildtype and Δ*nrdM* strains of A) SM43 and B) SO48 performed in Todd-Hewitt Broth, in biological duplicate. OD600 readings were performed every hour for 8 hrs. Mean and SD indicate. Growth of NrdM-I and NrdM-C alleles in C) SM43 and D) SO48 strain backgrounds when grown in streptococcal defined medium supplemented 5% v/v human serum. Serum growth was performed in biological triplicate and OD600 readings were taken at 0, 3, 6, and 24 hrs. Mean and SEM indicated.
