# Supplementary material for: Comparative Genomics of Streptococcus oralis Identifies Large Scale Homologous Recombination and a Genetic Variant Associated with Infection
Source: mSphere. 2022 Nov 2;7(6):e00509-22. doi: 10.1128/msphere.00509-22 (PMC9769543; doi:10.1128/msphere.00509-22)
Supplement: TABLE S3 [file msphere.00509-22-s0003.pdf]

Table S3. Primers used in this study

Primer	Sequence (5’-3’)	Use
43_A1 F	TTGTGAAAATCCCTAGAACG	SM43 upstream of *nrdM* region amplification
43_K1 F	AACCTTATGCAACGTTAAGAAAAAGAGTGG	SM43 downstream of *nrdM* region amplification for clean deletion
43_D1 O F	GAAAACTATCAACTGACAGC	SM43 upstream of *nrdM* for screening
43_D2 F	ATCCAGATAAAATTTACCAAGC	Internal primer of SM43 *nrdM*
43_I1 F	CGACATTGATCTAACCAAAACC	SM43 upstream of *nrdM* region outside recombination arms
43_K1 R	TTTTCTTAACGTTGCATAAGGTTCTCC	SM43 upstream of *nrdM* region amplification for clean deletion
43_A1 R	TTGTTACCTTAGTTAAAAGAATAGG	SM43 downstream of *nrdM* region amplification
43_D2 O R	CCTCAACTGTACAAAAAGC	SM43 downstream of *nrdM* for screening
43_D1 R	GATAAGAGATATAGTGTTCTGC	Internal primer of SM43 *nrdM*
43_I1 R	CAAGGCAGACTTTGATGC	SM43 downstream of *nrdM* region outside recombination arms
48_A F	GCAAGAATTTTTGTGACAAGG	SO48 upstream of *nrdM* region amplification
48_K F	CCTTATGCAACGTTAAGAAAAAGAGTGG	SO48 downstream of *nrdM* region amplification for clean deletion
48_D1 O F	GAAAACTATCAACTGACAGC	SO48 upstream of *nrdM* for screening
48_D2 F	AAACGTAATGGAGAAATTGC	Internal primer of SO48 *nrdM*
48_I F	TAAAATCAATAAAGAGAGCTACGG	SO48 upstream of *nrdM* region outside recombination arms
48_K R	TTTTCTTAACGTTGCATAAGGTTCTCC	SO48 upstream of *nrdM* region amplification for clean deletion
48_A R	GGTTGACTTTTCTTTCTGAATTAG	SO48 downstream of *nrdM* region amplification
48_D2 O R	CCTCAACTGTACAAAAAGC	SO48 downstream of *nrdM* for screening
48_I F	TAAAATCAATAAAGAGAGCTACGG	Internal primer of SO48 *nrdM*
48_I R	AGCTATCCACCCAACCG	SO48 downstream of *nrdM* region outside recombination arms
1492R	CGGCTACCTTGTTACGACTT	16S rRNA universal primer
8F	AGAGTTTGATCCTGGCTCAG	16S rRNA universal primer

UpRep_R	ATTACTTGCATAAGGTTCTCCTTTATTCTTTG	Amplifies upstream fragment of *nrdM*
Rep_F	AGGAGAACCTTATGCAAGTAATCAAACG	Amplifies *nrdM* gene
Rep_R	CACTCTTTTTCTTAACGGATTTGTTCAAAATG	Amplifies *nrdM* gene
DwnRep_F	AATCCGTTAAGAAAAAGAGTGGGAT	Amplifies downstream fragment of *nrdM*
GyrB_F	CAAGGTTTCCGTACAGC	Amplifies *gyrB*
GyrB_R	GCTTCTTGGAGTTTAATTTCTTG	Amplifies *gyrB*
